# Editorial: Measurement Invariance

**DOI:** 10.3389/fpsyg.2015.01064

**Published:** 2015-07-28

**Authors:** Rens Van De Schoot, Peter Schmidt, Alain De Beuckelaer, Kimberley Lek, Marielle Zondervan-Zwijnenburg

**Affiliations:** ^1^Department of Methods and Statistics, Utrecht UniversityUtrecht, Netherlands; ^2^Optentia Research Program, Faculty of Humanities, North-West UniversityVanderbijlpark, South Africa; ^3^Department of Political Sciences, University of GiessenGiessen, Germany; ^4^International Laboratory of Social-Cultural Research, Research University Higher School of EconomicsMoscow, Russia; ^5^Nijmegen School of Management, Institute for Management Research, Radboud University NijmegenNijmegen, Netherlands; ^6^School of Sociology and Population Studies, Renmin University of ChinaBeijing, China; ^7^Department of Personnel Management and Work and Organizational Psychology, Ghent UniversityGhent, Belgium

**Keywords:** measurement invariance, confirmatory factor analysis, Bayesian models, questionnaires, approximate measurement invariance, partial measurement invariance

Multi-item surveys are frequently used to study scores on latent factors, like human values, attitudes, and behavior. Such studies often include a comparison, between specific groups of individuals or residents of different countries, either at one or multiple points in time (i.e., a cross-sectional or a longitudinal comparison or both). If latent factor means are to be meaningfully compared, the measurement structures of the latent factor and their survey items should be stable, that is “invariant.” As proposed by Mellenbergh (1989), “measurement invariance” (MI) requires that the association between the items (or test scores) and the latent factors (or latent traits) of individuals should not depend on group membership or measurement occasion (i.e., time). In other words, if item scores are (approximately) multivariate normally distributed, conditional on the latent factor scores, the expected values, the covariances between items, and the unexplained variance unrelated to the latent factors should be equal across groups.

Many studies examining MI of survey scales have shown that the MI assumption is very hard to meet. In particular, strict forms of MI rarely hold. With “strict” we refer to a situation in which measurement parameters are exactly the same across groups or measurement occasions, that is an enforcement of zero tolerance with respect to deviations between groups or measurement occasions. Often, researchers just ignore MI issues and compare latent factor means across groups or measurement occasions even though the psychometric basis for such a practice does not hold. However, when a strict form of MI is not established and one must conclude that respondents attach different meanings to survey items, this makes it impossible to make valid comparisons between latent factor means. As such, the potential bias caused by measurement non-invariance obstructs the comparison of latent factor means (if strict MI does not hold) or regression coefficients (if less strict forms of MI do not hold).

Traditionally, MI is tested for in a multiple group confirmatory factor analysis (MGCFA) with groups defined by unordered categorical (i.e., nominal) between-subject variables. In MGCFA, MI is tested at each constraint of the latent factor model using a series of nested (latent) factor models. This traditional way of testing for MI originated with Jöreskog (1971), who was the first scholar to thoroughly discuss the invariance of latent factor (or measurement) structures. Additionally, Sörbom (1974, 1978) pioneered the specification and estimation of latent factor means using a multi-group SEM approach in LISREL (Jöreskog and Sörbom, 1996). Following these contributions the multi-group specification of latent factor structures has become widespread in all major SEM software programs (e.g., AMOS Arbuckle, 2006, EQS Bender and Wu, 1995, LAVAAN Rosseel, 2012, Mplus Muthén and Muthén, 2013, STATA STATA, 2015, and OpenMx Boker et al., 2011). Shortly thereafter, Byrne et al. (1989) introduced the distinction between full and partial MI. Although their introduction was of great value, the first formal treatment of different forms of MI and their consequences for the validity of multi-group/multi-time comparisons is attributable to Meredith (1993). So far, a tremendous amount of papers dealing with MI have been published. The literature on MI published in the 20th century is nicely summarized by Vandenberg and Lance (2000). Noteworthy is also the overview of applications in cross-cultural studies provided by Davidov et al. (2014), as well as a recent book by Millsap (2011) containing a general systematic treatment of the topic of MI. The traditional MGCFA approach to MI-testing is described by, for example, Byrne (2004), Chen et al. (2005), Gregorich (2006), van de Schoot et al. (2012), Vandenberg (2002) and Wicherts and Dolan (2010). Researchers entering the field of MI are recommended to first consult Meredith (1993) and Millsap (2011) before reading other valuable academic works.

Recent developments in statistics have provided new analytical tools for assessing MI. The aim of this special issue is to provide a forum for a discussion of MI, covering some crucial “themes”: (1) ways to assess and deal with measurement non-invariance; (2) Bayesian and IRT methods employing the concept of approximate MI; and (3) new or adjusted approaches for testing MI to fit increasingly complex statistical models and specific characteristics of survey data.

## Dealing with measurement non-invariance

If the test for MI indicates that strict MI across groups or time is not established, no sound psychometric basis is provided for the comparison of latent factor means. The absence of such psychometric basic is the first topic dealing with measurement non-invariance. A nice example of a situation in which such psychometric basis is absent is provided in the paper by Lommen et al. (2014). These authors show that comparing posttraumatic stress in soldiers before and after war-zone related traumatic events (the wars in Afghanistan or Iraq) is virtually impossible due to instability in thresholds. For a researcher this conclusion may be hard to digest, especially if the success of the study relies entirely on the possibility to make such meaningful comparisons over time. Within the context of their study the authors recommend considering pre- and post-symptom scores as representing separate constructs.

In the same vein, a failure to establish less strict forms of MI may be worrisome if meaningful comparisons of structural relationships between latent factor means are important to the study (e.g., the comparison of the magnitude of a correlation, regression, or path coefficient across groups/time). Hox et al. (2015), show how the non-establishment of less strict forms of MI can (partly) be explained and corrected for. They show that, in the context of mixed-mode surveys, non-invariance can be the effect of selection or measurement differences due to mode (e.g., web survey, telephone survey, face-to-face interview).

Detecting non-invariant items is the next topic dealing with measurement non-invariance. In the contribution of de Roover et al. (2014) a method is proposed based on cluster-wise simultaneous component analysis (SCA). Their method aims at detecting non-invariant items. Barendse et al. (2014) examined a Bayesian restricted (latent) factor analysis (RFA) method for the same purpose, namely detecting items violating the MI assumption. They concluded that Bayesian RFA methods are especially suited for detecting measurement bias.

Our special issue also contains a discussion on the importance of understanding whether the presence of (in)correctly specified factorial invariance parameters influences the assessment of other factor model parameters (e.g., intercepts, error variances, latent factor variances, and latent factor means). In a simulation study, Guenole and Brown (2014) investigated whether ignoring the non-invariant underlying structure of the latent factor leads to substantial regression parameter bias in categorical item factor analyses (CIFA). The authors urge researchers to avoid ignoring sources of non-invariance in CIFA when non-invariance occurs in *both* loadings and thresholds even if this occurs in only one item.

## Approximate measurement invariance

A relatively new research avenue in the MI literature deals with the use of Bayesian structural equation models (BSEM) to relax strict forms of MI (see Muthén and Asparouhov, 2012). In particular, exact zero constraints on the cross-group differences between all relevant measurement parameters (e.g., factor loadings and item intercepts) are substituted by “approximate” zero constraints. Instead of forcing item intercepts to be exactly equal across groups, a substantive prior distribution (around zero) is used to bring the parameters closer to one another, while allowing for some “wiggle room.” If there are many small differences between the groups in terms of intercepts or factor loadings, approximate MI seeks a balance between adherence to the requirements of MI, making comparisons possible, and obtaining a well-fitting model (i.e., a model that is more realistic given the data at hand). When the classical MI tests do not hold given the data, approximate MI represents a promising (and more realistic) alternative; the cross-group differences between all relevant measurement parameters are “hopefully” close enough to zero to allow making meaningful latent factor mean comparisons.

A tutorial paper introducing the method of approximate MI is presented by van de Schoot et al. (2013). Further, our special issue contains empirical examples comparing the results of Bayesian approximate MI to the results of the more traditional ways of MI-testing as applied to specific questionnaires: e.g., the Portrait Values Questionnaire, using data from the European Social Survey including data on many countries and many time points (Cieciuch et al., 2014; Zercher et al., 2015), the Hedonic and Eudaimonic Motives for Activities scale (Bujacz et al., 2014), and the Golombok-Rust Inventory of Marital State (Chiorri et al., 2014).

Furthermore, our special issue contains two extensions of approximate MI to the field of IRT (see also Fox and Verhagen, 2010). Instead of using substantive prior distributions as in the Bayesian approximate MI method, the method described by Fox establishes a measurement scale across countries and conceptualizes country-specific non-invariance in item parameters as random deviations through country-specific random item effects. In such conceptualization cross-group comparisons can still be made even in the presence of non-invariant items. Kelcey et al. (2014) developed a method based on Fox's approximate MI approach which is applicable whenever measurements are nested within raters and cross-classified among, for instance, countries. Another contribution to our special issue by Muthén and Asparouhov (2014) concerns the use of the *alignment* method (see also Asparouhov and Muthén, 2014) in IRT models, a method which is essential when applying approximate MI. This method minimizes a loss function which makes sure that there are a few large non-invariant measurement parameters instead of many smaller non-invariant measurement parameters, an optimal alignment strategy which resembles the rationale underlying rotation of factor solutions in EFA.

## Testing for MI in increasingly complex statistical models

For some complex statistical models, the traditional multi-group (MGCFA) approach to MI-testing has to be adjusted to meet the specific requirements of the data and/or the model. Examples of such adjustments can be found in our special issue. An assumption embedded within many methods to test for MI is that the grouping (i.e., auxiliary) variable is unordered (i.e., nominal). Wang et al. (2014) present a method to test for MI in cases in which the auxiliary variable is ordered or continuous. Verdam and Oort (2014) illustrate MI-testing for Kronecker restricted SEM models, which constitute parsimonious models that provide an alternative to longitudinal latent factor models. Adolf et al. (2014) examine MI in the context of multiple-occasion and multiple-subject time series models. In such models, MI has to be established (a) over time within subjects, (b) over subjects within occasions, and (c) over time and subjects simultaneously. Boom (2014) investigated MI in the context of children's development of increasingly advanced strategies over time, in for instance the way they deal with mathematical problems (e.g., strategies on how children learn to multiply numbers below 10). The use of different strategies is scored as a variable and development is seen as the movement from one strategy to a more advanced one and Boom shows how MI plays a crucial role when analyzing such data. Jak (2014) uses a multi-level framework and proposes an extension to the SEM framework, moving from models describing two-level data to models describing three-level data. Within this framework MI invariance can be tested across level 2 as well as across level 3 clustering variables.

Another application of MI finds its origin in multi-trait multi-method models (MTMM; Eid and Diener, 2006), in which multiple methods (or scales) and raters are used to quantify the set of latent factors under study. Geiser et al. (2014) demonstrate the advantage of moving from an exclusively covariance- or correlation-based MTMM approach to an approach that includes latent factor means. This approach results in more fine-grained information about convergent validity and method effects when testing for MI. Albeit being analyzed differently, a comparable design to the MTMM is the two-way rating design utilized in situations where subjects have to judge to what extent a particular scale or variable pertains to a particular concept or situation. Kroonenberg (2014) presents an approach applicable to the assessment of MI in two-way rating designs. In his approach, a hierarchy of models is proposed, each one conceptualizing a form of MI, varying in terms of strictness.

## Conclusion

Our special issue contains numerous simulation studies aiming at demonstrating the possibilities and limitations of different analytical tools to test for various forms of MI; tutorial papers providing the hands-on support needed when using the recent developed analytical tools to test for MI, as well as illustrations of how the analytical tools may be meaningfully applied in different fields of research when addressing issues related to MI across groups or time.

### Conflict of interest statement

The authors declare that the research was conducted in the absence of any commercial or financial relationships that could be construed as a potential conflict of interest.
